# First Case Report of Detection of Multidrug-Resistant *Enterobacter hormaechei* in Clinical Sample from an Aborted Ruminant

**DOI:** 10.3390/microorganisms10051036

**Published:** 2022-05-17

**Authors:** Sergey S. Zaitsev, Mariya A. Khizhnyakova, Valentina A. Feodorova

**Affiliations:** Federal Research Center for Virology and Microbiology, Branch in Saratov, 410028 Saratov, Russia; zaytsev-sergey@inbox.ru (S.S.Z.); khizhnyakova_mariya@mail.ru (M.A.K.)

**Keywords:** *Enterobacter cloacae* complex, *Enterobacter hormaechei*, multidrug-resistance, MDR, livestock, cattle, MLST, ST

## Abstract

The emergence of multidrug-resistant (MDR) bacterial strains is one of the significant global challenges with regard to bacterial drug-resistance control. *Enterobacter hormaechei* organisms belong to the *Enterobacter cloacae* complex (ECC) and are commonly recognized as causative agents for hospital infections. Recently, a few *E. hormaechei* MDR strains associated with infection in piglets, calves, and a fox were reported, highlighting the important role of animals and livestock in the emergence and spread of antimicrobial resistance. In this study, the vaginal swab sample from a 5-year-old cow with multiple anamnestic infectious abortions was carefully investigated. The animal was unresponsive to antibiotic therapy recommended by the veterinarian. The MDR bacterial strain isolated from the bovine sample, designated as the Saratov_2019, belonged to *Enterobacter hormaechei*. The genome-based phylogenetic analysis identified the isolate to be *Enterobacter hormaechei* subsp. *xiangfangensis*. The genome of the Saratov_2019 contained a 6364 bp plasmid. Importantly, we revealed the novel sequence type ST1416 and 13 MDR genes correlating with the MDR phenotype in only the chromosome but not the plasmid. These findings indicate that the potential spread of this strain may pose a threat for both animal and human health. The data obtained here support the notion of the important role of livestock in the emergence and spread of antimicrobial resistance, promoting careful investigation of the MDR spectra for livestock-related bacterial isolates. To the best of our knowledge, this is the first report on the association of *E. hormaechei* subsp. *xiangfangensis* with the infection of the reproductive system in cattle.

## 1. Introduction

The emergence and the increase in the finding of multidrug-resistant (MDR) strains is one of the current threats to both human and animal health worldwide [1]. According to the modern classification, MDR strains are non-susceptibility to at least one agent in three or more antimicrobial categories [2]. MDR strains are considered to be the most common causes of morbidity and mortality associated with infectious diseases [3,4,5]. In fact, certain MDR bacterial strains, which are currently known as ESKAPE organisms (*Enterococcus faecium*, *Staphylococcus aureus*, *Klebsiella pneumoniae*, *Acinetobacter baumannii*, *Pseudomonas aeruginosa*, and *Enterobacter* species) have been included in the WHO global priority pathogens list [6]. These bacterial agents demonstrate resistance to the majority of antibiotics including carbapenems, third-generation cephalosporins, peptide antibiotics, β-lactams, macrolides, and fluoroquinolones, and can cause untreatable severe and often fatal infections such as bloodstream infections and pneumonia. Successful therapy remains challenging and entails the assessment of novel and powerful antibiotics [6].

As members of the ESKAPE group, *Enterobacter* spp. has been reported to be one of the leading causes of MDR hospital infections [7]. *Enterobacter* spp. organisms belong to the family *Enterobacteriaceae* and are recognized as common causative agents in a variety of bloodstream and intraabdominal infections [8]. Currently, seven species are comprised in the genus *Enterobacter* (the *Enterobacter cloacae* complex (ECC)) such as *Enterobacter asburiae*, *Enterobacter carcinogenus*, *Enterobacter cloacae*, *Enterobacter hormaechei*, *Enterobacter kobei*, *Enterobacter nimipressuralis*, and *Enterobacter mori* [9]. Importantly, ECC strains belonging to at least two sequence types (STs), ST78 and ST171, have been recognized as emergent, MDR, and capable of being widespread [10]. Moreover, recently, the *E. hormaechei* isolate demonstrated a hypervirulent phenotype that was comparable with a hypervirulent *K. pneumoniae*-type strain in a *Galleria mellonella*-infection model [11].

Initially, these ESKAPE pathogens were associated with the most recurrent global cause of hospital infections. However, the emergence of MDR strains in animal husbandry indicates the important role of livestock in the emergence and spread of antimicrobial resistance (AMR) and highlights the necessity of the accurate identification of species, subspecies and spectra of AMR and MDR for outbreak-related isolates [12,13,14] as part of the global strategy to reduce the risk of emergence, spread, and food-borne transmission of MDR strains [14]. Recently, it was reported that *Enterobacteriaceae* isolated from farm animals may serve as reservoirs of AMR genes [15]. However, only limited information is available on the MDR isolates derived from either animal husbandry or wild-life animals. In fact, only a few ECC strains, namely *E. hormaechei*, have recently been identified as the causative agents of uterine infection in a dead fox [16], diarrhea in piglets [17], and respiratory disease in unweaned calves in China [18].

This study aimed to investigate the *E. hormaechei* subsp. *xiangfangensis* strain Saratov_2019 with MDR to eight groups of tested antibiotics, which was isolated from a cow with a reproductive system infection. Typically, *Enterobacter* spp. obtained from animals possess limited resistance to carbapenems [19], and to our knowledge, the *E. hormaechei* subsp. *xiangfangensis* with such an extensive MDR phenotype has not been previously found in cattle.

## 2. Materials and Methods

### 2.1. Specimens from a Cow

The vaginal, blood, and uretus swab samples (*n* = 3) for the study were obtained in April 2019 from a 5-year-old cow with multiple anamnestic infectious abortions. The vaginal swab was collected from a cow after thoroughly cleaning the vulva. The specimens were submitted to the diagnostic laboratory of the Saratov State Agrarian University in order to identify the possible cause of infection. The animal was kept at a small farm in the Saratov Region, Russia. The accompanying report for these animal specimens described the clinical signs of bovine genital tract inflammation such as mucopurulent or purulent vaginal discharge, fever, decreased milk yield, reduced appetite, tiredness, no sign of estrus, and several miscarriages in the past two years. The veterinarian who treated this cow reported that antibiotic therapy including oxytetracycline and third-generation cephalosporins had failed to improve the animal’s health. Thus, the animal was unresponsive to the antibiotic therapy recommended by the veterinarian. These clinical samples were studied bacteriologically using Endo agar (Becton Dickinson, Heidelberg, Germany), and cultivated at 37 °C in an aerobic environment for 3 days, which resulted in the isolation of the bacterial strain from the vaginal specimen only.

### 2.2. Determination of AMR Phenotype

The bacterial culture was screened for 12 antibiotics of several groups using the disk diffusion test (DDT) [20]. The test-panel of antibiotics included penicillins (Amoxicillin/Clavulanic acid; Pfizer, Inc. USA; Amoxicillin Trihydrate/Colistin Sulfate; Trionis, Russia), third-generation cephalosporins (Ceftriaxone; Biocom, Russia), fourth-generation cephalosporins (Cefquinome; Intervet International B.V., Netherlands), third-generation fluoroquinolone (Enrofloxacin; Bayer, Germany), oxyquinoline (Nitroxoline; Biosintez, Russia), tetracyclines (Oxytetracycline; Nita-farm, Russia), first-generation aminoglycoside (Kanamycin; PJSC “Krasfarma”, Russia), carbapenems (Meropenem; PJSC “Krasfarma”, Russia), lincosamides (Lincomycin; Velpharm, Russia), macrolides (Azithromycin; Beleka, Belarus), and nitroimidazole (Metronidazole; Nita-farm, Russia). Antibiotic sensitivity was interpreted in accordance with the Clinical and Laboratory Standards Institute (CLSI) [20].

### 2.3. DNA Extraction and Sequencing

The DNA from the bacterial isolate derived from the corresponding vaginal sample was extracted using the DNeasy Blood and Tissue Qiagen Kit (Qiagen, Hilden, Germany), and concentrations were measured with a spectrophotometer (BioRad Laboratories, Redmond, WA, USA). To preliminarily identify the type of microorganism, the isolated DNA from the bacterial culture was amplified by the 16S rRNA method followed by sequencing, as described previously [21]. The whole genome sequencing procedure of the extracted DNA was performed with the help of an Illumina HiSeq 2500 platform (Genoanalytica, Moscow, Russia, https://www.genoanalytica.ru/ (accessed on 15 May 2022)) and MinION (Oxford Nanopore Technologies, Oxford, UK). The DNA library preparation for Nanopore sequencing was undertaken with the 1D Genomic DNA by Ligation (SQK-LSK109) protocol (Oxford Nanopore Technologies, Oxford, UK) including DNA end repair, dA-tailing, and DNA clean-up steps. The final DNA library was sequenced using a FLO-MIN-106 R9.4 flow cell and the MinKNOW software (https://nanoporetech.com/community (accessed on 15 May 2022)).

### 2.4. Bioinformatic Data Processing

The preliminary DNA identification of the bacterial isolate was conducted through an analysis of the 16S rRNA gene amplicon sequencing and the EzBioCloud database (https://www.ezbiocloud.net (accessed on 15 May 2022)) for subspecies-level identification of the relevant microorganism. Hybrid assembly *de novo* was generated with Unicycler v 0.4.7 (https://github.com/rrwick/Unicycler (accessed on 15 May 2022)). Contig alignments were performed using Mauve software (http://darlinglab.org/mauve/mauve.html (accessed on 15 May 2022)). Primary metagenomic data analysis was carried out with the help of the Metagenomics Analysis Server MG-RAST (https://www.mg-rast.org (accessed on 15 May 2022)). Genome-based classification and identification were carried out with the help of the Type (Strain) Genome Server (https://tygs.dsmz.de/ (accessed on 15 May 2022)). The whole genome *de novo* assembled sequence data were deposited in the NCBI GenBank as *E. hormaechei* subsp. *xiangfangensis* strain Saratov_2019 (Acc. No. JAHFZP000000000.17).

### 2.5. MLST-Typing

The identification of allele profiles of multiple contigs and multi-locus sequence typing (MLST) was performed using a PubMLST database (https://pubmlst.org/ (accessed on 15 May 2022)). The ‘house-keeping genes‘-derived sequences were deposited in the PubMLST database (https://pubmlst.org/ (accessed on 15 May 2022)), with the access number—ST1416 (https://pubmlst.org/bigsdb?page=profileInfo&db=pubmlst_ecloacae_seqdef&scheme_id=1&profile_id=1416 (accessed on 15 May 2022)).

### 2.6. Determination of the AMR Genotype

The identification of antibiotic-resistance genes was carried out by using the RGI Resistance Gene Identifier (https://card.mcmaster.ca/analyze/rgi (accessed on 15 May 2022)).

### 2.7. List of Genomes Included in This Study

The following GenBank accession numbers were used for phylogenetic analysis in this study: NZ_JAHFZP000000000.1 (*E. hormaechei* subsp. *xiangfangensis* strain Saratov_2019), CP024908.1 (*E. hormaechei* subsp. *xiangfangensis* strain OSUKPC4_L), CP029246.1 (*E. hormaechei* subsp. *xiangfangensis* strain OSUVMCKPC4-2), CP043382.1 (*E. hormaechei* subsp. *xiangfangensis* strain WCHEX045001), CP023430.1 (*E. hormaechei* subsp. *xiangfangensis* strain UM_CRE-14), CP012165.1 (*E. hormaechei* subsp. *xiangfangensis* strain 34978), CP053103.1 (*E. hormaechei* subsp. *xiangfangensis* strain Ec61), NZ_CP017183.1 (*E. hormaechei* subsp. *xiangfangens* is strain LMG27195), CP010384.1 (*E. hormaechei* subsp. *xiangfangensis* strain 34399), NZ_CP061744.1(*E. hormaechei* strain NJGLYY90-CR), CP030007.1 (*E. hormaechei* subsp. *xiangfangensis* strain Pb204), NZ_CP017179.1 (*E. hormaechei* subsp. *steigerwaltii* strain DSM 16691), NZ_CP017180.1 (*E. hormaechei* subsp. *oharae* strain DSM 16687), NZ_CP017186.1 (*E. hormaechei* subsp. *hoffmannii* strain DSM 14563), NZ_MKEQ00000000.1 (*E. hormaechei* ATCC 49162), NZ_CP043318.1 (*Enterobacter chengduensis* WCHECl-C4), NZ_CP011863.1 (*Enterobacter asburiae* strain ATCC 35953), VTTY00000000.1 (*Enterobacter dykesii* strain E1), NZ_CP017184.1 (*Enterobacter roggenkampii* strain DSM 16690), LFDQ00000000.1 (*Enterobacter quasiroggenkampii* strain WCHECL1060), NZ_POVL00000000.1 (*Enterobacter sichuanensis* strain WCHECL1597), NZ_CP017181.1 (*Enterobacter kobei* strain DSM 13645), NZ_LT992502.1 (*Enterobacter bugandensis* isolate EB-247), NZ_QZCS00000000.1 (*Enterobacter. chuandaensis* strain 090028), NZ_MTFV00000000.1 (Enterobacter cloacae strain ATCC 13047), NZ_WJWQ00000000.1 (*E. cloacae* subsp. *dissolvens* ATCC 23373), QZCT00000000.1 (*Enterobacter huaxiensis* 090008), NZ_RXRX01000051.1 (*Enterobacter quasimori* 090044), SAMEA2548140 (*Enterobacter taylorae* NCTC 12126), NZ_FYBA01000003.1 (*Enterobacter cancerogenus* ATCC33241), PRJNA332046 (*E. kobei* ATCC BAA-260), NZ_BCTM00000000.1 (*Kluyvera cryocrescens* NBRC 102467), and BAFF01000003.1 (*Escherichia hermannii* NBRC 105704T).

## 3. Results and Discussion

A single vaginal swab yielded a bacterial strain that was identified by the 16S rRNA gene sequence on the EzBioCloud Server (https://www.ezbiocloud.net (accessed on 15 May 2022)). The results showed that the relevant microorganism was related to *Enterobacter hormaechei*. No microorganisms grew on the Endo agar plates from the blood and uretus specimens.

**Figure 1:**
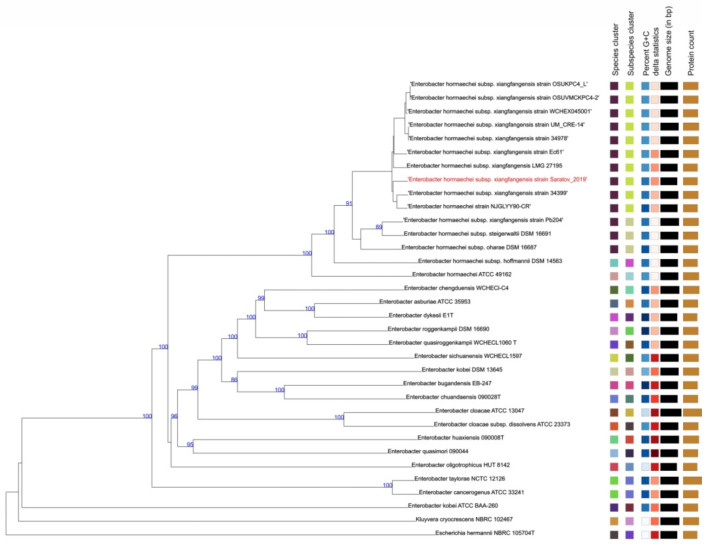
Phylogenetic analysis of the Saratov_2019 strain with regard to the different reference *Enterobacter* spp. genomes conducted with the use of the Type (Strain) Genome Server (TYGS, https://tygs.dsmz.de (accessed on 15 May 2022)). The color coding represents *Enterobacter* species and subspecies belonging to different clusters, G+C content, genome size, and protein count. The numbers on the branches show the distance between the taxa by delta statistics. The detailed information on the strains is presented in Table S1.

Overall, 67 contigs were assembled after next-generation sequencing (NGS) of the total DNA derived from the Saratov_2019 strain, of which 66 contigs belonged to the chromosomal DNA, and a single contig was identified as a circular plasmid replicon. The Genome BLAST Distance Phylogeny (GBDP) method based on the comparison of the Saratov_2019 strain with the TYGS database currently consisting of a comprehensive collection of 14,927 microbial type-strain genomes revealed that our strain had the highest homology (93.5%, CI 91.6–95.0) and formed a phylogenetic cluster with the reference strains *E. hormaechei* subsp. *xiangfangensis*, but not with the representatives of other subspecies of *Enterobacter* spp. (Figure 1). Notably, the plasmid identified in the strain designated as *E. hormaechei* Saratov_2019 (Acc. No. in NCBI NZ_JAHFZP010000042.1) with the size of 6364 bp showed high homology (98.84%) with the plasmid replicon pECL-90-4 that was recently (September, 2020) found in the strain *E. hormaechei* NJGLYY90-CR from China (Acc. No. in NCBI CP061745.1). The chromosomes of the strains Saratov_2019 and NJGLYY90-CR also demonstrated high homology (93%, CI 90.7–94.8), and these strains were located in close proximity on the phylogenetic tree (Figure 1). These data evidently indicate that the Saratov_2019 strain belongs to *E. hormaechei*. 

**Figure 2:**
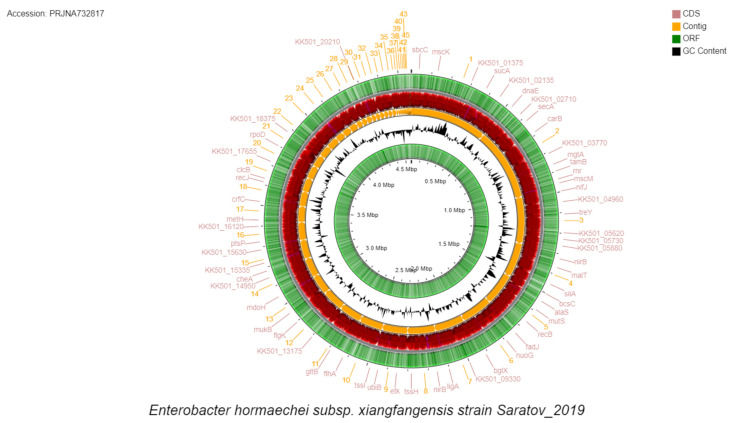
Graphic visualization of the *E. hormaechei* subsp. *xiangfangensis* Saratov_2019 genome after the automatic contig annotation that was generated based on the NCBI Prokaryotic Genome Annotation Pipeline (PGAP) (https://github.com/ncbi/pgap (accessed on 15 May 2022)). The pie chart (green) demonstrates the number of open reading frames detected. The red diagram shows the number of coding regions in the contigs. The inner chart (yellow) shows the number of contigs. The diagram (black color) shows the distribution of the GC-composition for 66 contigs of the strain. The contigs are located in ascending order of their length from the largest to the smallest.

The annotation of this strain from the *de novo* assembled whole genome, which was generated with the use of the NCBI Prokaryotic Genome Annotation Pipeline (PGAP) showed the presence of more than 4000 coding sequences (CDSs) and 77 pseudo genes as a result of either the frameshift mutations or premature stop codons in the relevant DNA sequences (Figure 2, Table S2).

Importantly, the Saratov-2019 strain had a novel sequence type (ST), ST1416 (Table 1). The relevant allelic profile consisted of the unique combination of the previously known allelic profiles of seven housekeeping genes: *dnaA*, *fusA*, *gyrB*, *leuS*, *pyrG*, *rplB*, and *rpoB* (https://pubmlst.org (accessed on 15 May 2022)).

**Figure 3:**
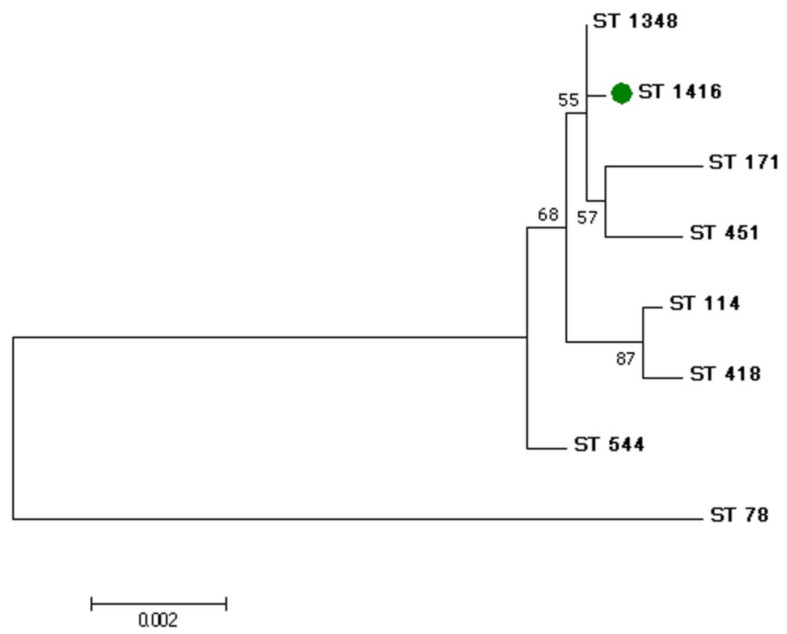
Phylogenetic tree of the strain Saratov_2019 ST1416 (labeled in green) based on a concatenate of the seven housekeeping genes *dnaA*, *fusA*, *gyrB*, *leuS*, *pyrG*, *rplB* and *rpoB* of *Enterobacter* (https://pubmlst.org (accessed on 15 May 2022)). The tree was constructed using the maximum likelihood method with MEGA 7.0 [27]. Bootstrap values = 100 (were shown at each node).

In fact, from three to four out of seven alleles of the Saratov_2019 strain were identical to those of the phylogenetically close reference *E. hormaechei* subsp. *xiangfangensis* strains (Figure 3, Table 1). These identical alleles were as follows: (i) *fusA*, *leuS* and *rplB* in the LMG27195 strain of ST544; (ii) *leuS*, *rplB* and *rpoB* in the strain 34399 of ST114; (iii) *fusA*, *gyrB* and *leuS* in the strains 34978, OSUKPC4_L, OSUVMCKPC4-2, UM_CRE-14, and WCHEX045001 of ST171; and (iv) *fusA*, *leuS*, *rplB* and *rpoB* in the strains Ec61 and NJGLYY90-CR of ST418 and ST451. Nevertheless, the *E. hormaechei* strain of ST1348 had an identical MLST profile in six alleles (4, 6, 19, 21, 44, and 46), and differed from Saratov_2019 by a single SNP for the allele 45 at position 48, which displayed a substitution C->T compared with that of allele 13 of Saratov_2019 corresponding to the *pyrG* gene (Figure 3, Table 1). Unfortunately, no information on the origin or even the name of this ST1348 isolate is available in the database. 

No identical alleles were found in the Saratov_2019 strain and the reference *E. hormaechei* strains of ST78 (Table 1), which together with ST171 was also identified as MDR [10]. In fact, ST78 formed a single branch separately from other STs. Both ST1416 and ST171 were found in the similar branch, although in different but closely related clades (Figure 3), meaning that these STs could have a single common ancestor.

To assess the AMR genotype-to-phenotype prediction of the Saratov_2019 strain, we investigated the relevant genomic data by the CARD service predicting drug-resistance genes. In parallel, the isolate was tested by the disk diffusion method to determine its susceptibility to different classes of antibiotics. The presence of the AMR genes for 16 different groups of antibiotics was predicted by the CARD for Saratov_2019 (Table 2).

At least 13 of them were successfully identified in the genome of this strain (Table 2). Interestingly, the Saratov_2019 strain demonstrated phenotypic resistance for the lincosamide group of antibiotics in DDT. However, it was not possible to identify the genetic determinism of resistance to this group of drugs for this strain. According to the CARD database, today, 38 ontology terms are currently known, which are associated with lincosamide resistance. We performed a comparative alignment of all 38 lincosamide resistance genes to identify a homologous sequence in the Saratov_2019 strain chromosome. Unfortunately, none of the annotated CDSs had homology with the relevant genes of thee CARD database. Perhaps the absence of lincosamide resistance genes could be explained by the incomplete assembly of the Saratov_2019 strain chromosome. The Saratov_2019 isolate demonstrated the presence of resistance to eight groups of antibiotics used in the diffusion test, indicating the actual MDR phenotype of this strain. No β-lactamase resistance genes or nine colistin resistance *mcr* gene variants (mcr-1–mcr-9) [24,28,29,30,31,32] were either predicted or detected during the genome annotation of both the chromosome and plasmid of the Saratov_2019 strain, which correlated with the observed sensitivity of this organism to the carbapenems and β-lactam antibiotics including the β-lactamase inhibitors. In fact, in DDT, the Saratov_2019 strain showed sensitivity to at least two groups of ß-lactam antibiotics, Cefquinome (fourth-generation cephalosporin) and Meropenem (synthetic antibiotic from the group of carbapenems). Thus, the Saratov_2019 strain was not a carbapenemase-producing *E. hormaechei* unlike the majority of the *E. hormaechei* spp. clinical isolates obtained from hospital infections worldwide [12,14,18,33,34,35,36].

## 4. Conclusions

According to our results, we report the first case of isolating the *E. hormaechei* subsp. *xiangfangensis* strain from a cow with a reproductive system infection. This MDR strain had a novel ST1416 and showed no carbapenem resistance. Future prospective research is critical to reveal the actual prevalence of MDR microorganisms in animal husbandry worldwide. A certain limitation of our study was that the panel of antibiotics used only the most common veterinary drugs. Additionally, we plan to significantly extend the number and species of animals investigated in our future research. Due to the potential risk to the health of both animals and humans who are professionally employed in animal husbandry, effective control over the spread of both the AMR and MDR bacterial strains has to be implemented.

## Figures and Tables

**Table 1:** Comparison of the allelic profiles of the seven housekeeping gene loci identified in the whole-genome assembly of the *E. hormaechei* subsp. *xiangfangensis* Saratov_2019 strain of ST1416 and *E. hormaechei* subsp. *xiangfangensis* reference strains* based on the phylogenetic analysis.

Strain ID	ST	Allele							Reference/Source
		*dnaA*	*fusA*	*gyrB*	*leuS*	*pyrG*	*rplB*	*rpoB*	
*E. hormaechei* subsp. *xiangfangensis* strain Saratov_2019	1416	46	21	19	44	13 ^a^	4	6	This study
Nameless ^b^	1348	46	21	19	44	45	4	6	https://pubmlst.org (accessed on 15 May 2022)
*E. hormaechei* subsp. *xiangfangensis* LMG27195	544	10	21	9	44	45	4	33	[22]
*E. hormaechei* subsp. *xiangfangensis* strain 34399	114	53	35	20	44	45	4	6	[23]
*E. hormaechei* subsp. *xiangfangensis* strain 34978	171	49	21	19	44	45	12	32	[24]
*E. hormaechei* subsp. *xiangfangensis* strain OSUKPC4_L	171	49	21	19	44	45	12	32	GenBank accession number: CP024908.1
*E. hormaechei* subsp. *xiangfangensis* strain OSUVMCKPC4-2	171	49	21	19	44	45	12	32	GenBank accession number: CP029246.1
*E. hormaechei* subsp. *xiangfangensis* strain UM_CRE-14	171	49	21	19	44	45	12	32	https://pubmlst.org (accessed on 15 May 2022)
*E. hormaechei* subsp. *xiangfangensis* strain WCHEX045001	171	49	21	19	44	45	12	32	https://pubmlst.org (accessed on 15 May 2022)
*E. hormaechei* subsp. *xiangfangensis* strain Ec61	451	146	21	148	44	99	4	6	[25]
*E. hormaechei* strain NJGLYY90-CR	418	53	35	154	44	45	4	6	[26]
*E. hormaechei* strain 1801 ^c^	78	8	9	6	9	9	6	8	https://pubmlst.org (accessed on 15 May 2022)

* The data are available in the PubMLST database (https://pubmlst.org/ (accessed on 15 May 2022)); ^a^ Different alleles of the gene in comparison with those in the Saratov_2019 strain are shown in red; ^b^ Information about the origin and characteristics of this strain are absent in the PubMLST database (https://pubmlst.org/ (accessed on 15 May 2022)); ^c^ the reference strain of 20 *E. hormaechei* strains of ST78 with identical allele profiles (the strains ID: 359, 362, 363, 372, 379, 380–383, 388, 391, 392, 401, 407–411, 446, and 491) present in the PubMLST database (https://pubmlst.org/ (accessed on 15 May 2022)).

**Table 2:** List of the AMR genes predicted and identified in the *E. hormaechei* subsp. *xiangfangensis* Saratov_2019 strain with the CARD service (https://card.mcmaster.ca/ (accessed on 15 May 2022)).

No.	Predicted with CARD			Identified in the Strain *				Confirmation by the DDT **		
	ARO Term ^a^	AMR Gene Family	Drug Group	Gene	Product	Locus_Tag in the Contig	Contig No.	Drug Group ^b^	Sensitive	Resistant
1	*Escherichia coli ampH* beta-lactamase	*ampC*-type beta-lactamase	C	*ampH*	D-alanyl-D-alaninecarboxypeptidase/endopeptidase AmpH	KK501_00085	1	C (third-generation)	-	+
2	*emrR*	major facilitator superfamily (MFS) antibiotic efflux pump	F	*emrR*	multidrug efflux transporter EmrAB transcriptional repressor EmrR	KK501_07160	5			
3	*rsmA*	resistance-nodulation-cell division (RND) antibiotic efflux pump	F, D	*rsmA*	16S rRNA (adenine(1518)-N(6)/adenine(1519)-N(6))- dimethyltransferase RsmA	KK501_03125	2			
4	*adeF*	resistance-nodulation-cell division (RND) antibiotic efflux pump	F, T	*oqxB*	multidrug efflux RND transporter permease subunit OqxB	KK501_09445	7			
5	*oqxA*	resistance-nodulation-cell division (RND) antibiotic efflux pump	F, G, T, D, Nf	*oqxB*	multidrug efflux RND transporter permease subunit OqxB	KK501_09445	7	T	-	+
6	*Klebsiella pneumoniae kpnE*	major facilitator superfamily (MFS) antibiotic efflux pump	M, Ag, T, P, R	*mdtJ*	multidrug/spermidine efflux SMR transporter subunit MdtJ	KK501_17290	19	First-generation Ag M	-	+ +
7	*Klebsiella pneumoniae kpnF*	major facilitator superfamily (MFS) antibiotic efflux pump	M, Ag, C, T, P, R	*mdtJ*	multidrug/spermidine efflux SMR transporter subunit MdtJ	KK501_17290	19			
8	*baeR*	resistance-nodulation-cell division (RND) antibiotic efflux pump	Ag, Ac	*baeR*	two-component system response regulator BaeR	KK501_18050	21			
9	*acrD*	resistance-nodulation-cell division (RND) antibiotic efflux pump	Ag	*acrD*	multidrug efflux RND transporter permease AcrD	KK501_09635	7			
10	*msbA*	ATP-binding cassette (ABC) antibiotic efflux pump	Nm	*msbA*	lipid A ABC transporter ATP-binding protein/permease MsbA	KK501_13920	13	Nm	-	+
11	*acrR*	AcrA/B complex	O	*acrR*	multidrug efflux transporter transcriptional repressor AcrR	KK501_00590	1	O	-	+
12	*fosA2*	fosfomycin thiol transferase	Ps	*fosA*	FosA/FosA2 family fosfomycin resistance glutathione transferase	KK501_03840	2	Ps	-	+
13	*Escherichia coli uhpT*	antibiotic-resistant UhpT	Ps	*uhpT*	hexose-6-phosphate: phosphate antiporter	KK501_18665	22	Ps	-	+

* Based on the annotation added by the NCBI Prokaryotic Genome Annotation Pipeline (PGAP); ** DDT, the disk diffusion test; ^a^ ARO, Antibiotic Resistance Ontology terms based on the CARD (https://card.mcmaster.ca (accessed on 15 May 2022)); ^b^ Ac, Aminocoumarins; Ag, Aminoglycosides; C, Cephalosporins; D, Diaminopyrimidines; F, Fluoroquinolone; G, Glycylcyclines; L, Lincomycins; M, Macrolides; Nf, Nitrofurans; Nm, Nitroimidazoles; O, Oxyquinolines; P, Peptide antibiotics; Pn, Phenicol antibiotics; Ps, Phosphonic antibiotics (Fosfomycins); R, Rifamycins; T, Tetracyclines.

## Data Availability

The data presented in this study are available in the open access databases NCBI GenBank and PubMLST.

## Supplementary Materials

The following supporting information can be downloaded at: https://www.mdpi.com/article/10.3390/microorganisms10051036/s1, Table S1: Brief characteristics of the *E. hormaechei* subsp. *xiangfangensis* Saratov_2019 after the automatic contig annotation that was generated based on the NCBI Prokaryotic Genome Annotation Pipeline (PGAP). Table S2: Brief characteristics of the *E. hormaechei* subsp. *xiangfangensis* Saratov_2019 after the automatic contig annotation that was generated based on the NCBI Prokaryotic Genome Annotation Pipeline (PGAP).
