# Supplementary material for: First Case Report of Detection of Multidrug-Resistant Enterobacter hormaechei in Clinical Sample from an Aborted Ruminant
Source: Microorganisms. 2022 May 17;10(5):1036. doi: 10.3390/microorganisms10051036 (PMC9145581; doi:10.3390/microorganisms10051036)
Supplement: Supplementary file 1 [file microorganisms-10-01036-s001.zip › microorganisms-1676177-supplementary.pdf]

Table S1. The detailed information about the strains used in the whole genome phylogenetic analysis after the automatic contig annotation that was generated based on the Type (Strain) Genome Server (TYGA) (https://tyga.dsmz.de).

Species cluster	Subspecies cluster	Species	Strain	Base pairs	Percent G+C, %	No. proteins
1	0	*Enterobacter quasimori*	090044	4 700 506	55,76	4384
5	3	*Enterobacter quasiroggenkampii*	WCHECL1060 T	4 805 203	55,69	4452
7	5	*Enterobacter chuandaensis*	090028T	4 629 218	55,68	4359
18	16	*Enterobacter chengduensis*	WCHECl-C4	5 123 725	55,74	4787
6	4	*Enterobacter sichuanensis*	WCHECL1597	4 869 039	55,24	4620
9	8	*Enterobacter hormaechei subsp. hoffmannii*	DSM 14563	4 678 566	55,33	4321
16	14	*Enterobacter roggenkampii*	DSM 16690	4 899 997	56,04	4474
19	17	*Enterobacter hormaechei*	ATCC 49162	4 802 284	55,24	4671
14	12	*Enterobacter kobei*	DSM 13645	4 927 478	54,91	4701
13	18	*Enterobacter hormaechei subsp. oharae*	DSM 16687	4 724 316	55,58	4436
13	18	*Enterobacter hormaechei subsp. steigerwaltii*	DSM 16691	4 782 480	55,55	4424
3	6	*Enterobacter taylorae*	NCTC 12126	4 928 119	55,73	6208
12	11	*Kluyvera cryocrescens*	NBRC 102467	5 044 663	53,85	4702
8	7	*Enterobacter bugandensis*	EB-247	4 717 613	56	4332
2	1	*Enterobacter dykesii*	E1T	4 509 323	55,85	4161
17	15	*Citrobacter youngae*	CCUG 30791	4 814 018	51,79	4556
11	10	*Enterobacter asburiae*	ATCC 35953	4 806 219	55,47	4426
4	2	*Escherichia hermannii*	NBRC 105704T	4 489 087	54,07	4160
10	9	*Enterobacter oligotrophicus*	HUT 8142	4 476 585	54,3	4142
3	6	*Enterobacter cancerogenus*	ATCC 33241	4 834 644	55,68	4518
15	13	*Enterobacter cloacae*	ATCC 13047	5 598 795	54,58	5518
13	19	*Enterobacter hormaechei subsp. xiangfangensis*	LMG 27195	4 661 849	55,28	4323
13	18	*Enterobacter hormaechei subsp. xiangfangensis*	Pb204	4 956 155	55,35	4599
13	19	*Enterobacter hormaechei strain*	NJGLYY90-CR	4 584 517	55,58	4267
13	19	*Enterobacter hormaechei subsp. xiangfangensis*	34399	4 784 288	55,31	4451

13	19	*Enterobacter hormaechei subsp. xiangfangensis*	34978	4 930 963	55,12	4626
13	19	*Enterobacter hormaechei subsp. xiangfangensis*	Ec61	4 887 670	55,28	4555
13	19	*Enterobacter hormaechei subsp. xiangfangensis*	LMG27195	4 661 849	55,28	4323
13	19	*Enterobacter hormaechei subsp. xiangfangensis*	OSUKPC4 L	4 753 669	55,28	4486
13	19	*Enterobacter hormaechei subsp. xiangfangensis*	OSUVMCKPC4-2	4 752 717	55,28	4483
13	19	*Enterobacter hormaechei subsp. xiangfangensis*	Saratov 2019	4 565 560	55,35	4308
13	19	*Enterobacter hormaechei subsp. xiangfangensis*	UM CRE-14	4 924 340	55,19	4679
13	19	*Enterobacter hormaechei subsp. xiangfangensis*	WCHEX045001	4 698 270	55,27	4377

Table S2. Brief characteristics of the *E. hormaechei subsp. xiangfangensis* Saratov_2019 after the automatic contig annotation that was generated based on the NCBI Prokaryotic Genome Annotation Pipeline (PGAP).

**Number of Genes**						**Number of Pseudo Genes**					**GC, %**
**Totally**	**CDSs**	**Coding proteins**	**ORFs**	**RNA**	**tRNAs**	**Totally**	**With frameshift**	**Incomplete**	**With internal stop**	**Multiple problems**	
4,405	4,239	4,239	22,979	89	78	77	33	30	20	6	55
